# Supervivencia de los pacientes con cáncer en Navarra y comparación con España

**DOI:** 10.23938/ASSN.1042

**Published:** 2023-08-16

**Authors:** Marcela Guevara, Miren Baztan, Rosana Burgui, Alberto Ovies, Aitziber Menéndez, Maribel Eciolaza, Conchi Moreno-Iribas, Eva Ardanaz

**Affiliations:** 1 Instituto de Salud Pública y Laboral de Navarra Departamento de Salud Gobierno de Navarra Pamplona España; 2 Centro de Investigación Biomédica en Red de Epidemiología y Salud Pública Madrid España; 3 Instituto de Investigación Sanitaria de Navarra Pamplona España

**Keywords:** Cáncer, Supervivencia, Pronóstico, Tendencias, Registro de Cáncer, Cancer, Survival, Prognosis, Trends, Cancer Registry

## Abstract

**Fundamento:**

Analizar la supervivencia de pacientes adultos diagnosticados de cáncer en Navarra, describir su tendencia y compararla con la supervivencia en España.

**Métodos:**

Los casos de personas adultas diagnosticadas de cáncer en los periodos 1999-2007 y 2008-2016 fueron seleccionados del registro poblacional de cáncer de Navarra; su estado vital se había actualizado hasta 2020. La supervivencia observada, la supervivencia neta (SN) y la SN estandarizada por edad (SNe) a cinco años, junto con sus intervalos de confianza al 95%, fueron estimados globalmente y para veintinueve grupos de cáncer.

**Resultados:**

Se analizaron 57.564 casos. La SNe de los hombres y mujeres diagnosticados en 2008-2016 fue 59,9% (59,1-60,8) y 63,8% (62,8-64,7), respectivamente. En hombres varió desde 13,4% (10,4-17,4) en cáncer de páncreas hasta 94,0% (88,1-100) en el de tiroides, y en mujeres desde 11,9% (7,2-19,7) en el cáncer de hígado hasta 95,6% (92,6-98,6) en el de tiroides. En comparación con los casos diagnosticados en 1999-2007, la SNe aumentó en diez grupos de cáncer, resultando un incremento global de 5,1 (4,1-6,0) puntos porcentuales. La SNe en Navarra fue 2,7 (1,9-3,4) puntos porcentuales mayor que la descrita en España en 2008-2013.

**Conclusiones:**

En Navarra la supervivencia de pacientes diagnosticados de cáncer en el periodo 2008-2016 mejoró significativamente respecto al periodo 1999-2007. Esta mejora obedece probablemente a múltiples factores, incluyendo diagnósticos más tempranos, opciones terapéuticas más efectivas y mejora del proceso asistencial. La supervivencia global fue mayor en las mujeres que en los hombres. Además, los resultados sugieren una supervivencia más alta en Navarra en comparación con España.

## Abstract (en)

**Background:**

To analyze the survival of adult cancer patients in Navarre, describe its trend, and compare the data for this Spanish Autonomous Community against that reported for Spain.

**Methods:**

Records of adult cancer patients were retrieved from the Navarre’s population-based cancer registry for two periods (1999-2007 and 2008-2016). The vital status had been updated to 2020. Observed survival, net survival and age-standardized net survival at five years with 95% confidence intervals were estimated overall and for twenty-nine cancer groups

**Results:**

We analyzed 57,564 cases. Age-standardized net survival was 59.9% (59.1-60.8) and 63.8% (62.8-64.7) for males and females diagnosed with cancer during the 2008-2016 period, respectively. Age-standardized net survival ranged from 13.4% (10.4-17.4) for pancreatic cancer to 94.0% (88.1-100) for thyroid cancer in male patients, and from 11.9% (7.2-19.7) for liver cancer to 95.6% (92.6-98.6-%) for thyroid cancer in female patients. Compared with cases diagnosed in the 1999-2007 period, age-standardized net survival increased in 10 cancer groups, resulting in an overall increase of 5.1 (4.1-6.0) percentage points. The age-standardized net survival in Navarre was 2.7 (1.9-3.4) percentage points higher than that described for Spain for the 2008-2013 period.

**Conclusions:**

In Navarre, the survival of cancer patients diagnosed during the 2008-2016 period improved significantly in comparison to the 1999-2007 period. Different factors may explain this improvement, including earlier diagnoses, more effective treatment options, and better healthcare processes. Overall, survival was higher in women than in men. Our results suggest a higher survival rate in Navarre than in Spain.

## INTRODUCCIÓN

El cáncer es una de las principales causas de morbimortalidad en el mundo. En Navarra, la incidencia media anual de cáncer en 2013-2016, excluyendo el de piel no melanoma, fue de 659 y 450 casos por cada 100.000 habitantes en hombres y mujeres, respectivamente^1^. En Navarra, al igual que en España, es la primera causa de muerte en hombres y la segunda en mujeres tras las enfermedades del sistema circulatorio^1^^,^^2^. El cáncer fue la causa del 47% de muertes prematuras (es decir, las ocurridas en menores de 75 años) en hombres y del 56% en mujeres en 2019^1^.

La supervivencia de los pacientes con cáncer es un indicador de gran interés tanto para pacientes como personal clínico y de gestión sanitaria, y está reconocida como un indicador esencial de la efectividad de los sistemas sanitarios en el control de esta enfermedad. En las últimas dos décadas en Navarra, se han iniciado o reforzado diferentes planes y programas de control del cáncer dirigidos a reducir la incidencia y mejorar la supervivencia y calidad de vida de los pacientes. En 2001 se inició el Plan Oncológico de Navarra, que fue evolucionando a lo largo de varios años e incluyó diversas acciones encaminadas a mejorar la calidad y accesibilidad de la atención oncológica, tales como el desarrollo de protocolos, implementación de circuitos rápidos de derivación ante sospecha fundada de cáncer, creación de equipos multidisciplinares de decisión terapéutica y creación de unidades asistenciales específicas^3^. El Plan de Salud de Navarra de 2006-2012 incluyó un plan estratégico para las enfermedades oncológicas^4^, en noviembre de 2013 comenzó la primera vuelta del programa de detección precoz de cáncer colorrectal^1^, y en 2016 se estableció la Estrategia de Prevención y Atención al Cáncer, que profundiza en un modelo de atención multidisciplinar e integrada^5^. Simultáneamente, durante estos años han surgido nuevos avances diagnósticos y terapéuticos que se han ido incorporando a la práctica clínica.

Se han descrito amplias diferencias en la supervivencia entre países y regiones^6^^-^^10^, por lo que es relevante estudiar la supervivencia a nivel de comunidad autónoma. El objetivo del presente estudio fue analizar la supervivencia de los pacientes adultos diagnosticados de cáncer entre 1999 y 2016 en Navarra, describir la tendencia por grupo de cáncer y comparar la supervivencia en Navarra con la informada por la Red Española de Registros de Cáncer (REDECAN)^11^.

## MÉTODOS

Este estudio se realizó utilizando datos anonimizados del Registro de Cáncer de Navarra, el cual, con fines de vigilancia en salud pública, recoge los casos incidentes en la población residente en Navarra (~640.650 habitantes en 2016). El registro de cáncer cumple con la normativa vigente en España, Ley Orgánica 3/2018 de Protección de Datos de Carácter Personal, y el Reglamento Europeo 2016/679. El cumplimiento de altos estándares de comparabilidad, exhaustividad y validez de sus datos está avalado por su inclusión en la publicación *Cancer Incidence in Five Continents*, de la Agencia Internacional de Investigación en Cáncer^12^.

Se seleccionaron los casos de personas adultas (15-99 años) diagnosticadas entre 1999 y 2016. Se incluyeron todos los tumores malignos invasivos de cualquier localización, excepto los de piel distintos de melanoma y, para mantener la comparabilidad con otros estudios, los tumores de comportamiento incierto e *in-situ* de vejiga. Se excluyeron los casos conocidos exclusivamente por el certificado de defunción y los diagnosticados incidentalmente en autopsia, debido a que se desconoce su tiempo de supervivencia.

La topografía y morfología de los tumores se codificó usando la tercera edición, primera revisión, de la Clasificación Internacional de Enfermedades para Oncología (CIE-O-3.1)^13^. Se aplicaron las reglas internacionales para el registro de neoplasias múltiples según la actualización contenida en la CIE-O-3.1^13^; los pacientes que tenían más de un cáncer primario se incluyeron en los análisis de cada cáncer. Las neoplasias hematológicas se agruparon de acuerdo con la clasificación de la OMS y las guías de HAEMACARE^14^^,^^15^. Los análisis se realizaron para el conjunto de todos los cánceres y por separado para 29 grupos de cáncer definidos por topografía y morfología. En la Tabla 1 se presentan los grupos de cáncer analizados y sus respectivos códigos de acuerdo con la Clasificación Internacional de Enfermedades, décima revisión (CIE-10).

**Tabla 1:** Grupos de cáncer analizados y sus correspondientes códigos de la Clasificación Internacional de Enfermedades-10ª revisión (CIE-10)

Grupo de cáncer	Códigos CIE-10	Grupo de cáncer	Códigos CIE-10
Cavidad oral y faringe	C01-C06, C09-C14	Próstata	C61
Esófago	C15	Testículo	C62
Estómago	C16	Riñón	C64
Colon	C18	Vejiga	C67, D090, D414
Recto	C19-C20	Encéfalo	C71
Hígado	C22	Tiroides	C73
Vesícula y vías biliares	C23-C24	Linfoma de Hodgkin	C81
Páncreas	C25	Linfomas no Hodgkin	C82-C86, C96
Laringe	C32	Mieloma	C90
Pulmón^#^	C33-C34	Leucemia linfoide aguda	C910
Melanoma de piel	C43	Leucemia linfoide crónica	C911
Mama femenina	C50	Leucemia mieloide aguda	C920, C923-C928, C930, C940-C946
Cuello de útero	C53	Leucemia mieloide crónica	C921
Cuerpo de útero	C54	Leucemias SAI y otras	C913-C919; C929; C931; C947; C950; C959
Ovario y anejos uterinos	C56, C570-C574, C577	Todos*	C00-C96 (excepto C44),
			D090, D414, D45-D47

SAI: *sine alter indication* (sin otra especificación); #: el grupo denominado “pulmón” incluye pulmón, bronquios y tráquea; *: excepto cáncer de piel distinto de melanoma.

El estado vital de los pacientes se había actualizado hasta el 31 de diciembre de 2020 a través de múltiples fuentes de información, principalmente el Registro de Mortalidad de Navarra, el Índice Nacional de Defunciones y las bases de datos de la tarjeta sanitaria, de la seguridad social y de la historia clínica informatizada.

Como indicadores de calidad de los datos se evaluó la proporción de los casos conocidos exclusivamente por el certificado de defunción, los verificados microscópicamente, los registrados con morfología inespecífica y los perdidos en el seguimiento^16^.

### Análisis estadístico

Las variables categóricas se describieron con frecuencia y porcentaje, y la edad con la mediana y el rango intercuartílico.

Los datos de dos periodos de diagnóstico, 1999-2007 y 2008-2016, se analizaron separadamente. Se estimó la supervivencia observada (SO) y neta (SN) a los cinco años del diagnóstico de cáncer mediante los métodos de Kaplan-Meier y de Pohar-Perme^17^, respectivamente. La SN representa la probabilidad de sobrevivir tras un tiempo dado desde el diagnóstico en ausencia de otras causas de muerte, para lo que se utiliza la mortalidad de la población general. A partir de los datos de población y defunciones por todas las causas del Instituto Nacional de Estadística, se construyeron Tablas de vida con las tasas de mortalidad de Navarra por sexo, año de edad y año calendario, suavizadas por el método de Elandt-Johnson^18^. Usamos el enfoque clásico de *cohorte* para estimar la supervivencia de los pacientes diagnosticados en el primer periodo, ya que tenían un seguimiento potencial mayor de cinco años. Para el segundo periodo, dado que los pacientes diagnosticados en 2016 tenían al cierre del estudio (final de 2020) un seguimiento potencial de entre cuatro y cinco años, usamos el enfoque *completo*, variante del de cohorte, que permite estimar la supervivencia de pacientes que han sido diagnosticados más recientemente, usando toda la información disponible incluyendo la de pacientes que no pudieron completar los cinco años de seguimiento^19^.

Con el fin de realizar comparaciones entre periodos y con otros estudios, se calculó también la SN estandarizada por edad (SNe) usando las poblaciones estándar internacionales de pacientes con cáncer (*International Cancer Survival Standards*, ICSS)^20^. Los grupos de edad usados en la estandarización fueron por tanto los de ICSS, que son 15-44, 45-54, 55-64, 65-74 y 75 o más años en todos los cánceres, salvo el de próstata en el que son 15-54, 55-64, 65-74, 75-84 y 85 o más años^20^. Cuando el número de casos en un grupo de edad fue insuficiente para la estimación, concretamente cuando a los cinco años no quedaban casos a riesgo, se unieron los datos de ese grupo de edad a uno adyacente y el estimador combinado se asignó a ambos grupos de edad antes de la estandarización^6^. La SNe no se calculó cuando en más de un grupo de edad el número de casos fue insuficiente para la estimación. Los intervalos de confianza del 95% (IC95%) se calcularon a partir de los errores estándar mediante la fórmula de Greenwood^21^. Para ver el cambio entre los dos periodos, se calculó la diferencia absoluta de la SNe, es decir: SNe _*2008-2016*_ - SNe _*1999-2007*_ . Los IC95% de la diferencia se calcularon asumiendo una distribución normal^22^, y se consideró un cambio como estadísticamente significativo si el IC95% de la diferencia no incluía el valor cero.

Para la comparación con España usamos las últimas cifras de SNe publicadas por REDECAN, las cuales se basaron en los datos disponibles de los registros poblacionales, que cubren el 26% de la población española^11^. Dicho estudio utilizó la misma metodología del presente estudio, y corresponde a los pacientes diagnosticados en 2008-2013, por lo que, para esta comparación, estimamos la SNe de Navarra del mismo periodo diagnóstico y calculamos la diferencia absoluta, SNe _*Navarra*_ - SNe _*España*_ , con sus IC95%.

Los análisis se realizaron con STATA 15.1 (*Stata Corporation, College Station*, TX, USA), usando el comando “stns” para estimar la SN.

## RESULTADOS

Se incluyeron 57.564 casos de cáncer diagnosticados en el periodo 1999-2016. El 60,1% eran hombres; la mediana de edad fue 70 años (61-77) en hombres y 67 (54-79) en mujeres. En la Tabla 2 se presenta el número de casos incluidos y los indicadores de calidad de los datos según grupos de cáncer. El 90,9% de los casos tenían verificación microscópica, si bien este porcentaje fue menor de 60% en el cáncer de hígado y de encéfalo. Sólo el 7,3% de los casos se registraron con una morfología inespecífica y menos de 0,2% fueron perdidos durante el seguimiento.

**Tabla 2:** Número de casos e indicadores de calidad de los datos según grupo de cáncer. Navarra, 1999-2016

Grupo de cáncer	Casos elegibles	Excluidos (%)		Incluidos	Indicadores de calidad de los datos (%)		
	N	SCD	Autopsia	N (%)	Verif. microsc	Morf. inesp.	Perd. seguim.
Cavidad oral y faringe	1.093	0,46	0,18	1.086 (99,36)	98,25	1,75	0,37
Esófago	552	1,63	0,36	541 (98,01)	95,01	4,81	0,18
Estómago	2.311	1,38	0,17	2.275 (98,44)	94,95	5,01	0,13
Colon	5.539	1,01	0,22	5.471 (98,77)	93,31	6,56	0,18
Recto	2.774	0,25	0,11	2.764 (99,64)	97,11	2,97	0,14
Hígado	1.186	2,87	1,1	1.139 (96,04)	54,08	6,41	0,09
Vesícula y vías biliares	800	0,88	1,25	783 (97,88)	72,54	20,82	0
Páncreas	1.724	1,97	1,04	1.672 (96,98)	69,32	30,68	0
Laringe	930	0,54	0,11	924 (99,35)	98,48	1,41	0,11
Pulmón	6.245	1,27	0,56	6.131 (98,17)	87,57	11,87	0,08
Melanoma de piel	1.457	0,14	0	1.455 (99,86)	99,79	0	0,14
Mama femenina	6.236	0,48	0,02	6.205 (99,5)	98,58	1,53	0,35
Cuello de útero	308	0,32	0	307 (99,68)	100	0	0,65
Cuerpo de útero	1.458	0,27	0,14	1.452 (99,59)	97,93	1,65	0,48
Ovario y anejos uterinos	792	0,76	0	786 (99,24)	91,48	8,4	0,13
Próstata	7.299	1	0,56	7.185 (98,44)	89,84	8,24	0,19
Testículo	269	0	0	269 (100,00)	99,26	0,74	0
Riñón	1.481	0,81	0,74	1.458 (98,45)	84,09	11,73	0,27
Vejiga	4.866	0,33	0,06	4.847 (99,61)	96,22	3,75	0,1
Encéfalo	1.081	1,39	0,28	1.063 (98,33)	57,67	11,76	0,09
Tiroides	1.141	0,35	2,28	1.111 (97,37)	99,82	0,36	0,54
Linfoma de Hodgkin	353	0,28	1,42	347 (98,30)	100	0	0,29
Linfomas no Hodgkin	1.700	0,41	0,71	1.681 (98,88)	97,68	8,03	0,18
Mieloma	602	1,5	0,5	590 (98,01)	95,25	0	0
Leucemia linfoide aguda	67	1,49	0	66 (98,51)	98,48	0	0
Leucemia linfoide crónica	561	0,53	0	558 (99,47)	95,34	0	0,36
Leucemia mieloide aguda	385	0,78	1,04	378 (98,18)	97,09	0	0
Leucemia mieloide crónica	100	1	0	99 (99,00)	98,99	0	0
Leucemias SAI y otras	178	6,74	1,12	164 (92,13)	93,29	35,98	0
Otros cánceres*	4.892	2,45	0,31	4.757 (97,24)	85,54	13,73	0,23
Todos*	58.380	1,01	0,39	57.564 (98,60)	90,92	7,29	0,19

SCD: casos conocidos solo por el certificado de defunción; Autopsia: casos diagnosticados en autopsia; Verif. microsc.: casos verificados microscópicamente; Morf. inesp.: morfología inespecífica correspondiente a los códigos de la Clasificación Internacional de Enfermedades para Oncología, 3.ª edición, 8000-8005 (tumores sólidos) y 9590, 9591, 9800, 9801, 9805, 9820, 9832 o 9860 (neoplasias hematológicas); Perd. seguim.: perdidos durante el seguimiento, son casos censurados vivos antes de los cinco años desde el diagnóstico, salvo que hayan sido censurados por finalización del seguimiento (31 de diciembre de 2020); SAI: *sine alter indication* (sin otra especificación); *: excepto cáncer de piel distinto de melanoma.

### Supervivencia a 5 años de los pacientes diagnosticados en 2008-2016

A los cinco años, los hombres diagnosticados en 2008-2016 presentaron una SO de 51,4% (IC95%: 50,7-52,1), una SN de 58,4 (IC95%: 57,6-59,3) y una SNe de 59,9% (59,1-60,8) en el total de cánceres. En las mujeres, las cifras de supervivencia a los cinco años fueron superiores: SO= 58,9% (58,0-59,7), SN= 62,9% (62,0-63,9) y SNe= 63,8% (62,8-64,7) (Fig. 1).

En los hombres, las SNe más bajas (<20%) se observaron en los cánceres de páncreas, encéfalo, esófago y pulmón, y las más altas (>80%) en los de tiroides, testículo, próstata, melanoma de piel y linfoma de Hodgkin (Fig. 1A, Tabla 3). En las mujeres, las supervivencias más bajas (<20%) se registraron en los cánceres de hígado, páncreas, esófago y encéfalo, y las más altas (>80%) en los de tiroides, melanoma de piel, linfoma de Hodgkin, cáncer de mama y leucemia linfoide crónica (Fig. 1B, Tabla 3).

**Figura 1A:**
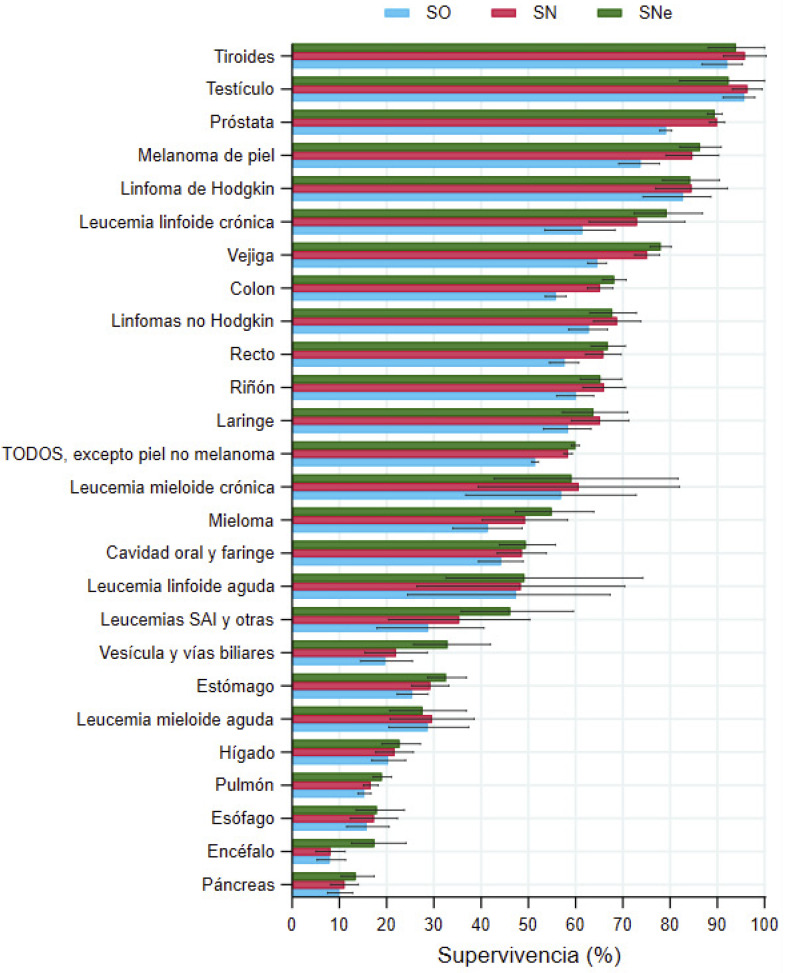
Hombres. Supervivencia observada (SO), neta (SN) y neta estandarizada por edad (SNe) a cinco años, con sus intervalos de confianza del 95%, en personas diagnosticadas de cáncer en Navarra en 2008-2016.

**Figura 1B:**
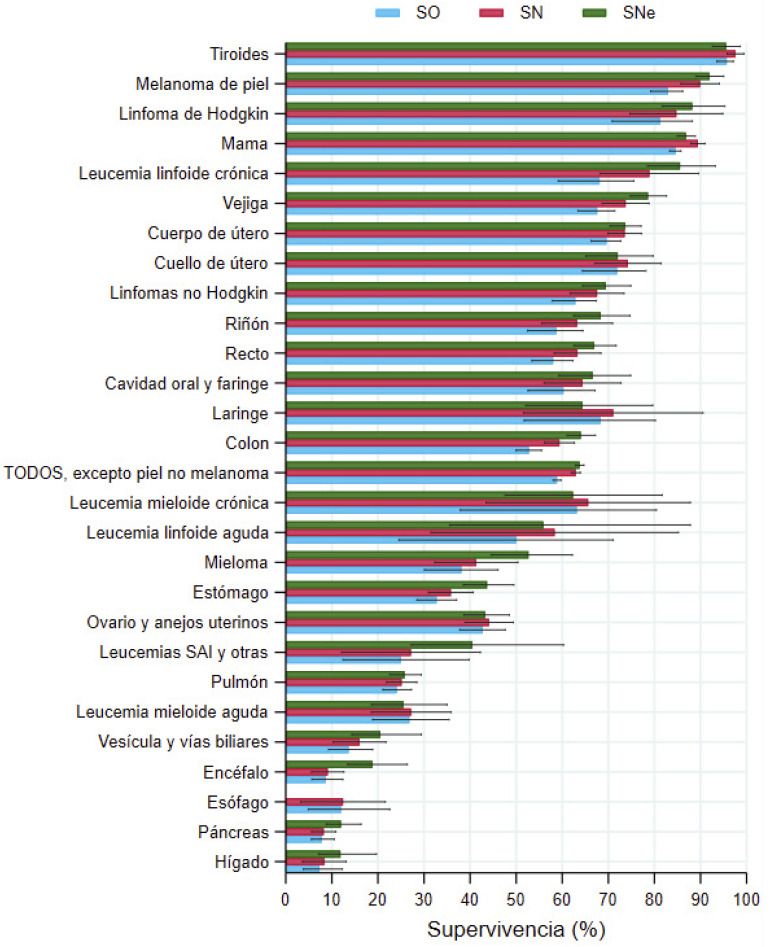
Mujeres. Supervivencia observada (SO), neta (SN) y neta estandarizada por edad (SNe) a cinco años, con sus intervalos de confianza del 95%, en personas diagnosticadas de cáncer en Navarra en 2008-2016.

**Tabla 3:** Supervivencia neta a cinco años estandarizada por edad (SNe) en hombres y mujeres diagnosticados de cáncer en Navarra en 2008-2016

	Hombres			Mujeres		
	N	SNe		N	SNe	
		%	IC 95%		%	IC 95%
Cavidad oral y faringe	433	49,5	43,9; 55,7	174	66,6	59,3; 74,9
Esófago ^a,b^	255	17,9	13,6; 23,7	50	NC	NC
Estómago	690	32,6	28,7; 36,9	456	43,7	38,6; 49,5
Colon	2.004	68,2	65,7; 70,7	1.270	64,1	61,1; 67,2
Recto	991	66,8	63,3; 70,5	473	67	62,5; 71,7
Hígado	488	22,7	19,0; 27,1	148	11,9	7,2; 19,7
Vesícula y vías biliares	204	32,9	25,7; 42,0	197	20,5	14,3; 29,4
Páncreas	496	13,4	10,4; 17,4	455	12	8,8; 16,4
Laringe ^a^	372	63,8	57,3; 71,0	41	64,4	52,1; 79,6
Pulmón	2.670	19	17,1; 21,1	736	25,8	22,6; 29,4
Melanoma de piel	407	86,3	82,1; 90,8	454	92	89,0; 95,0
Mama femenina	-	-	-	3.372	86,9	84,9; 88,9
Cuello de útero	-	-	-	165	72	65,1; 79,7
Cuerpo de útero	-	-	-	792	73,7	70,4; 77,1
Ovario y anejos uterinos	-	-	-	389	43,3	38,6; 48,5
Próstata	4.045	89,5	87,9; 91,0	-	-	-
Testículo ^c^	163	92,4	82,0; 100,0	-	-	-
Riñón	604	65,2	61,0; 69,7	258	68,3	62,5; 74,7
Vejiga	2.253	78	75,8; 80,2	530	78,6	74,8; 82,6
Encéfalo ^c^	317	17,4	12,6; 24,1	272	18,8	13,5; 26,4
Tiroides	164	94	88,1; 100,0	540	95,6	92,6; 98,6
Linfoma de Hodgkin	110	84,2	78,4; 90,4	80	88,2	81,7; 95,3
Linfomas no Hodgkin	534	67,8	63,0; 72,9	394	69,5	64,4; 74,9
Mieloma	174	54,9	47,3; 63,8	141	52,7	44,6; 62,2
Leucemia linfoide aguda ^c^	19	49,2	32,6; 74,2	16	55,9	35,6; 87,8
Leucemia linfoide crónica	169	79,3	72,4; 86,9	128	85,6	78,5; 93,2
Leucemia mieloide aguda	108	27,6	20,6; 36,9	110	25,5	18,6; 35,0
Leucemia mieloide crónica ^c^	28	59,1	42,8; 81,7	19	62,3	47,6; 81,7
Leucemias SAI y otras ^a^	59	46,1	35,8; 59,5	36	40,5	27,2; 60,3
Todos*	19.193	59,9	59,1; 60,8	12.788	63,8	62,8; 64,7

NC: no calculado; SAI: *sine alter indication* (sin otra especificación); **a**: se unieron los datos de los grupos de edad de 15-44 y 45-54 años y se asignó el estimador combinado a ambos grupos antes de la estandarización, debido a insuficiente número de casos para la estimación en uno de estos grupos en hombres o mujeres; **b**: no se calculó la SNe para el cáncer de esófago en mujeres porque en más de un grupo de edad el número de casos fue insuficiente para la estimación; la SN sin estandarizar fue 12,4% (IC95%: 3,2-21,6); **c**: se unieron los datos de los grupos de edad de 65-74 y ≥75 años y se asignó el estimador combinado a ambos grupos antes de la estandarización, debido a insuficiente número de casos para la estimación en uno de estos grupos en hombres o mujeres; *: excepto cáncer de piel distinto de melanoma.

**Figura 2:**
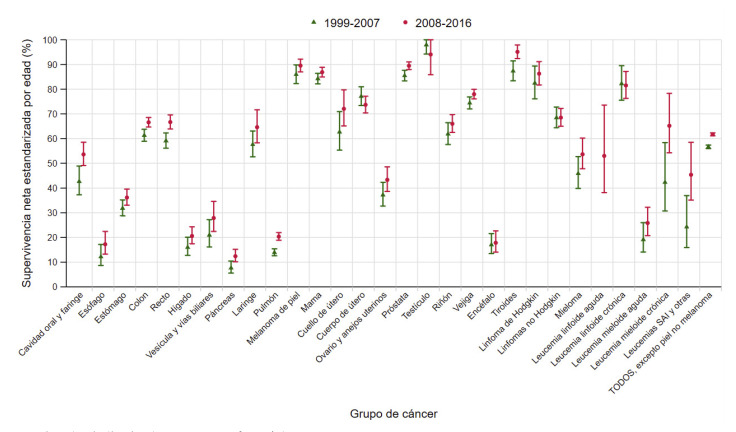
Supervivencia neta a cinco años estandarizada por edad, con sus intervalos de confianza del 95%, en pacientes de ambos sexos diagnosticados de cáncer en Navarra en 1999-2007 y 2008-2016.

SAI: *sine alter indication* (sin otra especificación).

**Figura 3:**
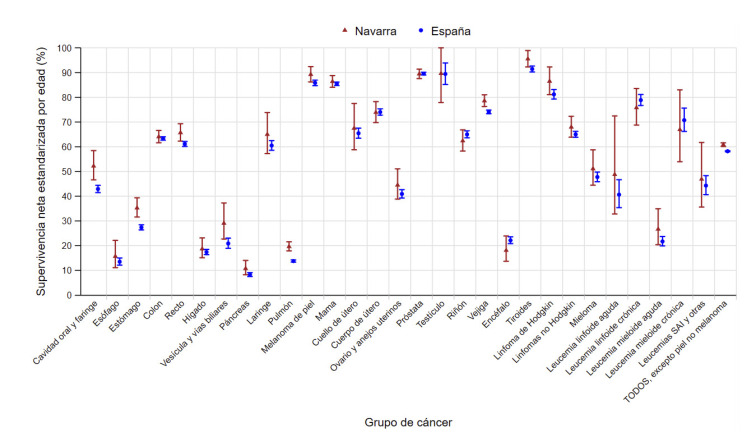
Supervivencia neta a cinco años estandarizada por edad, con sus intervalos de confianza del 95%, en los pacientes diagnosticados de cáncer en Navarra y en España en 2008-2013, en ambos sexos. Datos de España obtenidos de la Red Española de Registros de Cáncer (REDECAN)^11^.

SAI: *sine alter indication* (sin otra especificación).

La SNe global fue 3,8 (2,6-5,1) puntos porcentuales más alta en las mujeres que en los hombres. La mayor ventaja femenina se encontró en el cáncer de cavidad oral y faringe (SNe= 66,6%; IC95%: 59,3-74,9 frente a 49,5%; IC95%: 43,9-55,7). La supervivencia de las mujeres también fue mayor en los cánceres de estómago, pulmón y melanoma de piel, mientras que fue menor en el cáncer de hígado y en el de vesícula y vías biliares (Tabla 3).

**Tabla 4:** Supervivencia neta a cinco años estandarizada por edad (SNe, %) en pacientes de ambos sexos diagnosticados de cáncer en Navarra en 1999-2007 y 2008-2016, y cambio absoluto entre periodos

	1999-2007		2008-2016		Cambio absoluto	
	SNe	IC95%	SNe	IC95%	Diferencia^#^	IC95%
Cavidad oral y faringe	42,7	37,3; 48,9	53,6	49,1; 58,5	10,9	3,5; 18,3
Esófago^a^	12,2	8,7; 17,2	17,3	13,3; 22,5	5,1	−1,1; 11,2
Estómago	31,8	28,8; 35,2	36,2	33,1; 39,6	4,4	−0,2; 8,9
Colon	61,3	58,9; 63,8	66,6	64,7; 68,5	5,3	2,2; 8,4
Recto	59,1	56,1; 62,3	66,7	63,9; 69,6	7,6	3,4; 11,8
Hígado	16	12,8; 20,1	20,6	17,4; 24,3	4,6	−0,4; 9,6
Vesícula y vías biliares	21	16,2; 27,2	27,9	22,5; 34,6	6,9	−1,2; 15,1
Páncreas	7,7	5,6; 10,5	12,4	10,2; 15,2	4,8	1,3; 8,2
Laringe	57,6	52,6; 63,0	64,6	58,3; 71,6	7	−1,5; 15,5
Pulmón	13,9	12,6; 15,4	20,4	18,9; 22,0	6,4	4,3; 8,5
Melanoma de piel	85,9	82,2; 89,8	89,5	87,0; 92,1	3,6	−1,0; 8,2
Mama femenina	84,2	82,1; 86,4	86,9	84,9; 88,9	2,6	−0,3; 5,5
Cuello de útero	62,6	55,3; 70,9	72	65,1; 79,7	9,4	−1,3; 20,1
Cuerpo de útero	77,1	73,4; 81,0	73,7	70,4; 77,1	−3,4	−8,5; 1,7
Ovario y anejos uterinos	37,2	32,7; 42,3	43,3	38,6; 48,5	6,1	−0,8; 13,0
Próstata	85,5	83,3; 87,6	89,5	87,9; 91,0	4	1,4; 6,7
Testículo^b^	97,9	94,2; 100,0	94,1	85,9; 100,0	−3,9	−13,2; 5,5
Riñón	61,8	57,6; 66,4	66	62,5; 69,7	4,1	−1,6; 9,8
Vejiga	74,4	72,0; 76,8	78	76,1; 79,9	3,6	0,5; 6,7
Encéfalo^b^	17,1	13,5; 21,6	17,9	14,1; 22,7	0,8	−5,0; 6,7
Tiroides	87,3	83,4; 91,5	95,1	92,4; 97,9	7,8	2,9; 12,6
Linfoma de Hodgkin	82,4	76,1; 89,4	86,3	81,7; 91,1	3,8	−4,3; 12,0
Linfomas no Hodgkin	68,4	64,4; 72,7	68,5	65,0; 72,2	0,1	−5,5; 5,6
Mieloma	45,8	39,9; 52,7	53,6	47,8; 60,2	7,8	−1,1; 16,7
Leucemia linfoide aguda^b,c^	NC	NC	53	38,2; 73,5	NC	NC
Leucemia linfoide crónica	82,2	75,5; 89,5	81,5	76,2; 87,1	−0,7	−9,6; 8,1
Leucemia mieloide aguda^b^	19,1	14,1; 26,0	25,9	20,8; 32,2	6,7	−1,4; 14,9
Leucemia mieloide crónica	42,4	30,7; 58,4	65,2	54,2; 78,3	22,8	4,7; 40,9
Leucemias SAI y otras^a^	24,3	15,9; 37,0	45,3	35,1; 58,5	21,1	5,7; 36,5
Todos *	56,6	55,9; 57,4	61,7	61,1; 62,3	5,1	4,1; 6,0

#: Diferencia absoluta entre periodos en puntos porcentuales; SAI: *sine alter indication* (sin otra especificación); NC: no calculado; **a**: se unieron los datos de los grupos de edad de 15-44 y 45-54 años y se asignó el estimador combinado a ambos grupos antes de la estandarización, debido a insuficiente número de casos para la estimación en los más jóvenes; **b**: se unieron los datos de los grupos de edad de 65-74 y ≥75 años y se asignó el estimador combinado a ambos grupos antes de la estandarización, debido a insuficiente número de casos para la estimación en los mayores; **c**: no se calculó la SNe en la leucemia linfoide aguda del primer periodo porque en más de un grupo de edad el número de casos fue insuficiente para la estimación; la SN sin estandarizar fue 25,9% (IC95%: 11,0-40,9); *: excepto cáncer de piel distinto de melanoma.

### Tendencia de la supervivencia a 5 años entre los periodos diagnósticos 1999-2007 y 2008-2016

La SNe de las personas diagnosticadas en 2008-2016 aumentó 5,1 (4,1-6,0) puntos porcentuales en el conjunto de todos los cánceres respecto a las diagnosticadas en 1999-2007 (Tabla 4, Fig. 2). Por grupo de cáncer, hubo incrementos significativos de la SNe de más de 10 puntos porcentuales en la leucemia mieloide crónica, el grupo de leucemias sin otra especificación y otras, y el cáncer de cavidad oral y faringe. En los cánceres de colon, recto, pulmón y tiroides se observaron incrementos significativos de entre 5 y 10 puntos porcentuales, y en los de páncreas, próstata y vejiga, de entre 3 y 5 puntos porcentuales. Hubo además una mejora cercana a la significación estadística en los cánceres de estómago, hígado y mama femenina. No se observaron descensos significativos de la SNe en ningún grupo tumoral.

### Comparación de la supervivencia del cáncer en Navarra y España

Si comparamos las estimaciones más recientes publicadas de SNe a cinco años en España (periodo de diagnóstico 2008-2013)^11^ con las encontradas en Navarra en el mismo periodo (Fig. 3), la SNe para el total de cánceres fue más alta en Navarra que en España, con una diferencia de 2,7 (1,9-3,4) puntos porcentuales. Destacó la mayor SNe en Navarra respecto a España en los cánceres de cavidad oral y faringe (9,3 puntos porcentuales de diferencia; IC95%: 3,2-15,4), vesícula y vías biliares (8,2; IC95%: 0,7-15,7), estómago (7,9; IC95%: 3,9-11,9), y pulmón (5,9; IC95%: 4,0-7,8). También se observó una SNe más alta en Navarra que en España en el cáncer de recto, melanoma de piel, cáncer de vejiga y tiroides. En el resto de grupos tumorales no se detectaron diferencias estadísticamente significativas en la SNe.

## DISCUSIÓN

El presente estudio muestra una mejora de 5,1 puntos porcentuales en la supervivencia a 5 años de las personas adultas diagnosticadas de cáncer en 2008-2016 en Navarra, que alcanzó el 61,7%, en comparación con el 56,6% de los pacientes diagnosticados en 1999-2007. En la mayoría de los grupos de cáncer la supervivencia tendió al aumento, alcanzando la significación estadística en diez de ellos, incluyendo varios de los más frecuentes en la población. Mejoró significativamente la supervivencia en la leucemia mieloide crónica, el grupo de leucemias sin otra especificación y otras, en el cáncer de cavidad oral y faringe, en los de colon, recto, pulmón, tiroides, páncreas, próstata y vejiga.

En Navarra, varios planes y programas de control del cáncer se han implementado o reforzado en los últimos años, teniendo como uno de sus objetivos mejorar la supervivencia de los pacientes^3^^-^^5^. Por otra parte, algunos estudios poblacionales han demostrado una alta adherencia a las guías de práctica clínica en cáncer^23^^,^^24^. Todo ello, junto con los múltiples avances diagnósticos y terapéuticos que se han producido, hace que sean esperables mejoras de la supervivencia. Este estudio constata una mejora significativa entre los dos periodos analizados. Destaca la magnitud del incremento en la leucemia mieloide crónica y el grupo de leucemias sin otra especificación y otras, a lo que habrán contribuido los avances en el conocimiento biológico y en las técnicas citogenéticas, que han permitido mejorar la caracterización y tratamiento de las leucemias^25^. La leucemia mieloide crónica es la neoplasia para la que se ha logrado mayor mejora de la supervivencia en España en las últimas dos décadas^11^^,^^26^, lo que es atribuible principalmente a la introducción, a comienzos de los 2000, de la terapia dirigida con inhibidores de la tirosina quinasa que han mejorado considerablemente su pronóstico^27^.

La mejora que encontramos en la supervivencia en el cáncer de cavidad oral y faringe podría estar relacionada, entre otros factores, con mejoras en los tratamientos y con una posible disminución de la proporción de casos asociados al consumo de tabaco que a su vez se asocian con peor pronóstico^28^^,^^29^. Al aumento de la supervivencia en los cánceres de colon y recto puede estar contribuyendo el programa de cribado que inició a finales de 2013, tanto por mejora real de la supervivencia gracias a diagnósticos más tempranos, como por el sesgo de adelanto diagnóstico. La mejora en cáncer de pulmón es atribuible en gran parte a importantes avances en los métodos diagnósticos y los tratamientos^30^. En el cáncer de tiroides, una parte del aumento de la supervivencia puede corresponder a una mayor detección de tumores indolentes de bajo riesgo^31^. Por otra parte, no se observó mejora de la supervivencia en el cáncer de cuerpo uterino, hallazgo que coincide con lo encontrado en España y en otros países como EEUU y Francia, y que en buena medida refleja una falta de avances sustanciales en el tratamiento^11^^,^^26^^,^^32^^,^^33^.

La amplia variación de la supervivencia que encontramos entre grupos de cáncer es acorde con lo reportado en la literatura^6^^-^^8^^,^^11^^,^^26^. Los cánceres con peor pronóstico fueron los de páncreas, esófago, encéfalo, pulmón e hígado, los cuales en conjunto representaron el 22% de los casos en hombres y el 13% en mujeres. En el otro extremo, mostraron muy buen pronóstico los cánceres de tiroides, testículo, melanoma de piel, próstata, mama femenina, linfoma de Hodgkin y leucemia linfoide crónica, que supusieron el 26,4% de los casos en hombres y el 35,8% en mujeres.

En el conjunto de cánceres, la supervivencia fue más alta en las mujeres que en los hombres, consistente con la literatura^11^^,^^26^^,^^34^^-^^36^, si bien la diferencia absoluta que encontramos (3,8%) fue menor que la descrita en España (6,4%)^11^. Esta ventaja femenina se explica en buena medida por la diferente distribución de grupos tumorales por sexo, como se ha mencionado antes. Además, la supervivencia fue significativamente mayor en las mujeres que en los hombres para cuatro grupos tumorales: cavidad oral y faringe, estómago, pulmón y melanoma de piel, los cuales también en España y Europa han mostrado mejor pronóstico en las mujeres que en los hombres^7^^,^^11^^,^^29^. Esta diferencia fue especialmente marcada en el cáncer de cavidad oral y faringe (diferencia absoluta de 17,2%), consistente con lo descrito en España (19%)^11^ y en otros países^29^^,^^37^, y que posiblemente se relaciona en parte con una menor prevalencia de tabaquismo y consumo de alcohol y, por lo tanto, de comorbilidades entre las mujeres. Por el contrario, en el cáncer de hígado y el de vesícula y vías biliares se encontró una supervivencia mayor en los hombres que en las mujeres. Entre los posibles factores explicativos de las diferencias de supervivencia entre ambos sexos se encuentran factores biológicos, diferencias en la distribución de factores de riesgo, morfología y sub-localización tumorales, estadio al diagnóstico y comorbilidad^35^^,^^36^.

La SNe a cinco años de las personas diagnosticadas en 2008-2013 en Navarra fue 2,7 puntos porcentuales más alta que la descrita en España^11^, a expensas de una mejor supervivencia en ocho de los grupos tumorales analizados. Las cifras que observamos en Navarra se encuentran en el rango de las publicadas por el estudio ICBP SURVMARK-2, que presenta la supervivencia para siete cánceres en siete países de ingresos altos (Australia, Canadá, Dinamarca, Irlanda, Nueva Zelanda, Noruega y el Reino Unido)^8^. Hasta el momento no se ha publicado un estimador de la supervivencia para el total de cánceres en Europa de un periodo tan actual que sea comparable con el último periodo que analizamos; sin embargo, si comparamos la supervivencia que encontramos en Navarra en el primer periodo, 1999-2007, vemos que fue 3,4 y 2,4 puntos porcentuales más alta que la descrita en España y Europa, respectivamente, en 2000-2007^26^^,^^34^.

Entre las limitaciones del estudio cabe destacar que no se analizaron factores pronósticos importantes, como el estadio al diagnóstico o los tratamientos recibidos, que podrían haber ayudado a explicar las tendencias. A pesar de haber analizado periodos de nueve años, en algunos grupos tumorales el número de casos fue pequeño por lo que los resultados deben interpretarse con precaución. Las estimaciones de REDECAN, usadas en la comparación con España, incluyeron en su cálculo los datos de Navarra^11^. Entre las fortalezas del estudio están su ámbito poblacional, que minimiza el sesgo de selección, el uso de procedimientos de registro estándar y la alta calidad de los datos. Además, hay pocos estudios en la literatura que hayan incluido un número tan amplio de grupos de cáncer y, hasta donde sabemos, este es el que presenta datos más actuales. Nuestros resultados son útiles por tanto para países de nuestro entorno, donde las circunstancias que marcan las tendencias podrían ser similares.

En conclusión, observamos una mejora significativa de la supervivencia de los pacientes diagnosticados de cáncer en Navarra en 2008-2016 respecto a los diagnosticados en 1999-2007. Esta mejora probablemente obedece a múltiples factores, incluyendo diagnósticos más tempranos, opciones terapéuticas más efectivas y mejora del proceso asistencial. El desarrollo de planes específicos de lucha contra el cáncer probablemente ha contribuido a las mejoras descritas. La supervivencia global fue mayor en las mujeres que en los hombres. Por otra parte, este estudio sugiere una ventaja en la supervivencia de los pacientes en Navarra en comparación con la estimada en España. Para entender las diferencias en la supervivencia son necesarios estudios que incluyan las diferencias en estadio al diagnóstico y el manejo de los pacientes, entre otros factores.
